# Pennsylvania State Veterinary Medical Association

**Published:** 1893-10

**Authors:** Robert Gladfelter, W. Horace Hoskins

**Affiliations:** Recording Secretary; President


*Pennsylvania State Veterinary Medical Association.*—The semi-annual meeting of the Pennsylvania State Veterinary Medical Association was held in the parlors of the Y. M. C. Association building at Scranton, on September 5th, 1893. The meeting was called to order by the President, Dr. W. H. Hoskins, at ten o'clock A.M.

On the roll call the following members responded: Doctors Benner, DuBois, Gladfelter. J. R. Hart, Helmer, Hoskins, Hooker, Houldsworth, Miller, Pearson, Ridge, Thos. B. Rayner, Jas. B. Rayner, Stanton and Timberman.

The New Jersey Veterinary Medical Association was represented by delegates, Drs. Duston and Lockwood. Letters and telegrams were received from Mayor, Wm. L. Connell, Secretary of Agriculture Edge, and Drs. Zuill, Harger, and Robt. Ward, expressing their regrets for their inability to attend.

The President, Dr. W. H. Hoskins, then read his address. He called attention to the wonderful growth of the City of Scranton, which has practically developed from one industry alone. He impressed upon all the rapid growth in numbers and advance in value of the Veterinary Association throughout the country. and the importance of the coming 30th Annual Meeting of the United States Veterinary Medical Association, and the first Veterinary Congress of America. In severe language he referred to the infamous spoils system which has extended to the Bureau of Animal Industry, and laid the responsibility at the doors of the Veterinarians themselves; he hoped that they would apply the remedy in their own hands before this Bureau is endangered in its existence, even though it has been lessened in its value as a scientific body. He urged the members to use their best influence in guiding young men to the best Veterinary Schools, for the recent rapid multiplication of Veterinary Colleges is no proof of their need or surety of their ability and equipment to discharge their grave responsibilities.

The Secretary read the following list of applicants for membership: Drs. D. B. Fitzpatrick, W. L. Rhodes, H. B. Felton, J. B. Seitter, S. J. Nicholson, M. T. Miller, W. J. Tomlinson, R. J. Fox, A. O. Cawley. The following veterinarians made application at meeting: H. R. Church and H. L Marsack.

A recess was then held for the Board of Trustees to convene. There being three absentees on Board, the President appointed Drs. Helmer, Timberman and Thos. B. Rayner to fill the vacancies The Board of Trustees favorably recommended as follows: H. R. Church, D. B. Fitzpatrick, Howard B. Felton. Jos. B. Seittler, S. J. Nicholson, W. T. Miller, H. L. Marsack; they laid over for consideration Drs. Cawley. Fox and Tomlinson. and recommended that they be requested to be present at the next meeting of the Board of Trustees at Philadelphia, in March next. The recommendations of the Board of Trustees were approved, and the new applicants elected by the Secretary's ballot under a suspension of the rules.

The Corresponding Secretary, Dr. W. H. Ridge, gave a brief record of the work of the past six months, noting among other matter the action of Housmann and Dunn, instrument makers, of Chicago, in publishing Dr. S. J. J. Hargers' article read before this Association on Laryngotomy, without crediting the author with the same, or the Association to whom said paper was extended. He stated that our membership had increased by 40 under the present officers. making the total number to date 112, and further asked that each member bring a new one for our March meeting. The Secretary's report was received and a vote of thanks was extended to him.

The Legislation Committee made a brief report, and some discussion followed as to the wisdom of amending the present Act in regard to Veterinary registration. Delegates to our meeting were asked to address the members, and Dr. Duston of the New Jersey Veterinary Medical Association responded. He regretted that there was not more active, more harmonious work, and a stronger fraternal feeling among the profession generally.

Under the head of Intelligence and Education, reference was made to the Washington Veterinary College, which was continuing its short term of instruction, and was reported as having determined to run it as a money-making institution, and not in the behalf of higher veterinary education. On motion a Committee was appointed to draft suitable resolutions to the Washington College, and also to report in regard to the action of Housmann and Dunn. The Committee subsequently reported the following resolutions :—

*Whereas*, The almost unanimous sentiment of the Veterinary profession demands the professional schools to require of their students at least three years' attendance upon instructions, and to constantly increase their equipments as science demand ;

*And Whereas*, This Association hears with regret that the National College in Washington is to be continued as a two years short term school, with a poor and incomplete equipment ;

*Be it Resolved*, That this Association renews its condemnation of this school, which we think tends to lower and degrade the standard of Veterinary science.

LEONARD PEARSON,
W. B. E. MILLER,
JACOB HELMER,
*Committee.*

*Whereas*, The firm of Housmann, McComb and Dunn has printed without authority the article on "Roaring," prepared by Dr. S. J. Harger for this Association, and have used the said article as an advertisement for instruments manufactured by them, and without giving credit to the author or the Association for which it was prepared ;

*Be it Resolved*, That the attention of the Veterinary profession be called to this disreputable act by the publication of this preamble and resolution in the Veterinary Journals.

LEONARD PEARSON,
W. B. E. MILLER,
JACOB HELMER,
*Committee.*

Through the Corresponding Secretary, Dr. Thos B. Rogers offered a paper on castration. The Association, by vote, accepted the same, and accorded the author a vote of thanks.

The Treasurer made a verbal report, and suggested the purchase of a special book to keep accounts of members, and he was authorized to purchase the same.

At one o'clock P.M. the meeting adjourned for lunch. On reconvening at 2.15 P.M., Dr. Helmer read a valuable and suggestive paper on Inflammation of the Internal Structure of the Eye. This was followed by a paper by Dr. Leonard Pearson on "Anthrax," which had an added interest through his recent investigations of some serious outbreaks in this State and Delaware. Dr. T. H. Timberman, in a paper on "Punctured Wounds of the Foot," recorded some remarkably successful results when the bursar was involved, by the use of chlorid of zinc in mild solution, and careful dressing to close the wound from external influences. Dr. Miller kindly read the paper on castration contributed by Dr. Rogers. The author severely criticised the operation standing, and laid many schirrus cords at its doar. He referred to the false claims about "Cryptorchides," citing many cases where they could not be truly termed a ridgling, and it was no trouble to operate.

This was followed by Dr. Ridge on "Dystocia," and the employment of force in these cases. He recorded a number of experiences where extraordinary measures had been used to the great suffering of the animal, and always the loss of the young where they were offering such presentations as made birth impossible, and only the return of the fœtus, and its position changed to a normal one, brought an easy birth, and great relief to the parent. His paper was full of valuable practical suggestions.

The discussion of these papers, bringing many interesting points, was taken part in by Drs. Miller, Pearson, Kooker, Helmer, Timberman, Duston, Thos. B. Rayner, and others.

A resolution was then passed that the reports, papers, etc., be given to the "American Veterinary Review," and that arrangements be made in connection therewith for the printing of 350 copies of the same.

A vote of thanks was extended to the Wyoming Valley Veterinary Medical Association for their kindness in caring for the members and arranging for the meeting. A similar vote of thanks was given to those who had made reports and offered papers.

The meeting adjourned at six P. M. At eight P. M., through the courtesy of the Wyoming Valley Veterinary Medical Association, a trip was made into one of the coal mines, which was a pleasant experience to all who had the pleasure of availing themselves of the opportunity. The following morning at 8.30 the members were treated to a trip to Far View, ascending the mountain from Carbondale by a series of 14 plains. It proved a delightful day. A rich view of beautiful scenery, and many pleasant experiences long to be remembered.

ROBERT GLADFELTER,
*Recording Secretary.*

W. HORACE HOSKINS,
*President.*

